# Biomanufacturing of Tomato-Derived Nanovesicles

**DOI:** 10.3390/foods9121852

**Published:** 2020-12-11

**Authors:** Ramesh Bokka, Anna Paulina Ramos, Immacolata Fiume, Mauro Manno, Samuele Raccosta, Lilla Turiák, Simon Sugár, Giorgia Adamo, Tamás Csizmadia, Gabriella Pocsfalvi

**Affiliations:** 1 Extracellular Vesicles and Mass Spectrometry Group, Institute of Biosciences and BioResources, National Research Council of Italy, 80131 Naples, Italy; ramesh.chem2008@gmail.com (R.B.); a.paulina.ramos@gmail.com (A.P.R.); immacolata.fiume@ibbr.cnr.it (I.F.); 2 Institute of Biophysics, National Research Council of Italy, 90146 Palermo, Italy; mauro.manno@cnr.it (M.M.);; 3 MS Proteomics Research Group, Hungarian Academy of Sciences, Research Centre for Natural Sciences, 1117 Budapest, Hungary; liliat7@gmail.com (L.T.); sugarsimi@gmail.com (S.S.); 4 Institute for Biomedical Research and Innovation, National Research Council of Italy, 90146 Palermo, Italy; giorgia.adamo@gmail.com; 5 Department of Anatomy, Cell and Developmental Biology, Eötvös Loránd University, 1117 Budapest, Hungary; aldhissla1987a@gmail.com

**Keywords:** nanovesicles, tomato, size-exclusion chromatography, biomanufacturing, proteomics, phospholipids, *Solanum lycopersicum* L., gradient ultracentrifugation, differential ultracentrifugation

## Abstract

Micro- and nano-sized vesicles (MVs and NVs, respectively) from edible plant resources are gaining increasing interest as green, sustainable, and biocompatible materials for the development of next-generation delivery vectors. The isolation of vesicles from complex plant matrix is a significant challenge considering the trade-off between yield and purity. Here, we used differential ultracentrifugation (dUC) for the bulk production of MVs and NVs from tomato (*Solanum lycopersicum* L.) fruit and analyzed their physical and morphological characteristics and biocargo profiles. The protein and phospholipid cargo shared considerable similarities between MVs and NVs. Phosphatidic acid was the most abundant phospholipid identified in NVs and MVs. The bulk vesicle isolates were further purified using sucrose density gradient ultracentrifugation (gUC) or size-exclusion chromatography (SEC). We showed that SEC using gravity column efficiently removed co-purifying matrix components including proteins and small molecular species. dUC/SEC yielded a high yield of purified vesicles in terms of number of particles (2.6 × 10^15^ particles) and protein quantities (6.9 ± 1.5 mg) per kilogram of tomato. dUC/gUC method separated two vesicle populations on the basis of buoyant density. Proteomics and in silico studies of the SEC-purified MVs and NVs support the presence of different intra- and extracellular vesicles with highly abundant lipoxygenase (LOX), ATPases, and heat shock proteins (HSPs), as well as a set of proteins that overlaps with that previously reported in tomato chromoplast.

## 1. Introduction

The study of nanometer-sized vesicles (NVs) isolated from whole plants or plant organs has opened a new branch of research in the field of extracellular vesicles (EVs). [1,2,3] Several recent reports and review articles describe the successful isolation of NVs from a great variety of edible fruits and vegetables (Table 1) [1,2,3,4,5,6,7,8,9,10,11,12,13,14,15]. Plant-derived exosome-like NVs are morphologically similar to the small EVs (sEVs) or exosomes isolated from mammalian cell cultures and biofluids [16], and the methods used for their isolation and characterization are also the same or similar. Typically, homogenized plant is the starting material for the isolation of NVs, which is a very complex matrix that makes the isolation process very challenging. Plant-derived vesicle isolates are more complex than mammalian cell-derived EVs and contain both intra- and extracellular vesicles [7]. There is a growing interest in advanced strategies for the production of NVs from plant resources due to their numerous promising applications, especially in the nutraceutical [6], cosmeceutical [8], and therapeutic fields [9]. Due to their inherent role in intracellular trafficking, native NVs are efficiently taken up by recipient cells to which they transfer their lipids, mRNAs, microRNAs, and protein biocargo [10,11]. Interestingly, some native NVs have been shown to possess antitumor [12], anti-inflammatory [13,14,15,16], anti-aging [8], and anti-Alzheimer [17] properties. For example, Zhang et. al. reported that ginger-derived NVs reduce inflammation in inflammatory bowel disease and colitis-associated cancer in mice [13]. In another work, ginger-derived NVs were proven to be efficient against Alzheimer’s disease in a rat model [17]. Both grape- and broccoli-derived NVs were shown to inhibit colitis [14] and to protect from dextran sulfate sodium-induced colitis (DSS-induced colitis) [16] using mouse models. Moreover, turmeric (*Curcuma longa* L.)-derived NVs were shown to reduce colitis and promote intestinal wound repair [15]. Interestingly, NVs from the fruit juice of *Citrus limon* L. strongly suppressed tumor growth in rats [12]. NVs extracted from *Dendropanax morbifera* were shown to have strong inhibitory effect on melanin production in a human epidermis model, which promotes their future cosmeceutical applications [18]. The use of plant-derived NVs as novel drug delivery systems is boosted by their intrinsic resistance to the acidic gastric environment of the stomach [19], efficient uptake at target site, and low cost and sustainable production [18]. Moreover, NVs can be loaded with exogenous molecules such as drugs or other health-promoting substances or modified for engineered targeting. For example, Wang et al. loaded grapefruit-derived NVs with curcumin, folic acid, and zymosan A [19], and in another work ginger-derived NVs were loaded with doxorubicin anti-cancer drug [20].

EVs can be isolated in several ways. The applied methods rely on centrifugation, filtration, precipitation, and chromatography-based separations. Intense research is undertaken to make improvements in this field by optimizing traditionally used methods or by finding new ways. The ISEV community for the efficient separation of EVs suggests the gradient ultracentrifugation (gUC) method. gUC employs an inert gradient medium in which the EV-containing sample is centrifuged at high centrifugal force to reach the equilibrium isodensity zone. The method generally achieves good separation of particles of different densities and is able to separate EVs from the soluble smaller components and to resolve EV subpopulations differing in buoyant densities. There are a number of different gradient media for the separation of EVs—sucrose and iodixanol being the most popular ones. Recently, other methods, such as tangential flow filtration (TFF), [35] polymer-based precipitation, and size-exclusion chromatography (SEC) are gaining field for the isolation or second step purification of vesicles [36]. Amongst these, SEC is one of the best performing methods for EV separation/purification, especially from biological fluids [37] and in combination with ultrafiltration or ultracentrifugation. SEC uses porous beads to separate EVs from other biopolymers (proteins, polysaccharides, proteoglycans, etc.) and small molecules on the basis of their hydrodynamic volume [38]. The separation takes place during the filtration of a sample solution through a gravity or HPLC column containing the porous beads with radii smaller than the EVs [37]. SEC columns can be packed with different stationary phases including Sepharose 2B, Sepharose CL-4B, Sepharose CL-2B, and Sephacryl S-400 for the gravity-driven separation of exosome-like vesicles; however, commercially available, ready-made columns (IZON qEV and ExoSpin) with proprietary resin bed are also available and they respond well to the inter-laboratory reproducibility challenges. The separation efficiency of the column depends on the chemical composition and structure of the stationary phase [38]. SEC is relatively fast and reproducible, providing relatively high yields [37]. Moreover, SEC was shown to separate EVs from soluble smaller molecules without effecting the integrity and biological activity of the vesicles [39].

Isolation of exosome-like vesicles from complex plant matrices is very challenging. The different organs such as fruit, leaf, seed, and root have different physical structures and tissue types. As is shown in (Table 1), in the plant field, differential ultracentrifugation (dUC) is the most frequently used method today for the purification of EV-like vesicles. The main drawback of the dUC method in the isolation of NVs from the highly complex matrix is the low efficiency to separate the vesicles from the co-sedimenting broken cells, insoluble polymers from the extracellular matrix, cell wall, etc. This usually negatively influences not only the reproducibility but also the downstream analysis and their applications in biotechnology. The combination of dUC/gUC generally solves this problem and results in purer fraction than dUC alone. Thus far, dUC/gUC has only been limitedly applied in the plant field (Table 1) [40] because it is time-consuming and includes multiple washing and pelleting steps that can negatively affect the final vesicle yields. Other methods, such as polyethylene glycol precipitation, have also been employed for the purification of ginger rhizome-derived vesicles [22]. Even though the precipitation method is easy and does not require specialized equipment, it has some drawbacks such as co-purification of non-vesicular proteins and requirement of pre- and post-clean-up steps [22]. Ultrafiltration using membrane filters with defined molecular weight or size limits [25] has also been used in combination with dUC. Ultrafiltration is quick and easy but the applied force during filtration may lead to deformation and breaking of large vesicles [40].

Here, micro- (MVs) and nanovesicles (NVs) were isolated by dUC from tomato (*Solanum lycopersicum* L.) fruit. Tomato is the second most important vegetable crop after potato [41]. Tomato fruit has been extensively studied for its health benefits such as antioxidant activity associated with lycopene. Tomato fruit is a potentially high-value resource of vesicles to be applied in future functional foods. Vesicles isolated by dUC were characterized by dynamic light scattering (DLS), nanoparticle tracking analysis (NTA), transmission electron microscopy (TEM), sodium dodecyl sulphate polyacrylamide gel electrophoresis (SDS-PAGE), and thin-layer chromatography (TLC) to prove the vesicle character and the biocargo complexity of the isolates. Vesicles were further purified using two different methods: gUC, which separates the components on the basis of their buoyant density, and SEC, which separates according to their size. dUC/SEC and dUC/gUC methods were compared in their ability to purify tomato-derived vesicles in terms of yield, purity, and number of vesicles. Finally, nanoHPLC–MS/MS-based shotgun proteomics was performed on the SEC-purified vesicles to obtain information about the complexity of protein biocargo they carry.

## 2. Materials and Methods

### 2.1. Plant Material and Isolation of Vesicles by Differential Ultracentrifugation

Tomato fruits (Piccadilly) were purchased from the local market (G.M Fruit, Sicily, Italy). Tomatoes (500 g) were washed with Milli-Q water and put into boiling water for a few seconds to remove exocarp. Fruits were transferred to mixture grinder containing a 1:1 weight to volume ratio extraction buffer (pH 8) composed of 100 mM phosphate, 10 mM ethylenediamine tetraacetic acid (EDTA), and protease inhibitor cocktail (0.25 mL leupeptine (1 mg/mL), 1.25 mL 100mM phenylmethylsulfonyl fluoride (PMSF), and 0.8 mL 1M sodium azide). The sample was homogenized 3 times at maximum velocity for 10 s. Homogenized sample was subjected to the dUC protocol. Briefly, sequential low velocity centrifugations were performed at 400× *g*, 800× *g*, and 2000× *g* using a swinging-bucket rotor for 30 minutes for each step at 22 °C. Supernatant was centrifuged at 15,000× *g* in a fixed-angle rotor for 30 minutes at 22 °C to collect the pellet containing the microvesicle (MV) fraction. The supernatant was ultracentrifuged at 100,000× *g* for 120 minutes at 4 °C using a SW28 Beckman rotor in a Beckman Coulter Optima L-90K ultracentrifuge. Pellet was solubilized in a small amount of the extraction buffer and protein quantity was measured using the Qubit Protein Assay Kit (Thermo Fisher Scientific, Rockford, IL USA).

### 2.2. Gradient Ultracentrifugation

Gradient ultracentrifugation (gUC) was performed on NV-enriched samples isolated by dUC using a continuous sucrose gradient 8–45% (*w/v*) in a polypropylene ultracentrifugation tube (Beckman Coulter, Brea, CA, USA). Sample (5 mg measured as protein content) was dispersed in 500 µL of extraction buffer by agitation and pipetting, then centrifuged at 100,000× *g* for 2 h at 4 °C using an SW28Ti rotor (Beckman Coulter, Brea, CA, USA). Six fractions were collected from top to bottom as follows: Fr1, 5 mL; Fr2, 7 mL; Fr3 (Band 1) 7.5 mL; Fr4, 4.5 mL; Fr 5 (band2), 3 mL; and Fr6, 11 mL. Each fraction was washed to remove sucrose by using centrifugation at 100,000× *g* for 1 h at 4 °C in extraction buffer. The resulting pellets were suspended in small volumes of extraction buffer and protein quantity was measured. The experiment was performed three times using NVs from 3 different dUC extractions.

### 2.3. Size-Exclusion Chromatography

#### 2.3.1. Column Packing

The gravity size-exclusion chromatography column (SEC) was prepared using 15 mL of sepharose CL-2B (GE Healthcare, Cytiva, Uppsala, Sweden) particle size 60–200 μm, pore size 100,000–20,000,000 Da, and 2% cross-linked agarose gel filtration matrix. A total of 15 mL of the sepharose CL-2B ethanol suspension were equilibrated with phosphate-buffered saline (PBS; pH 7.4), which was previously degassed and filtered with a 0.22 µm filter (Millex-GP filter, Millipore, Burlington, MA, USA). Then, 10 mL of the sepharose CL-2B was packed in a 15 mL Chromabond column with a polyethylene (PE) frit integrated at the bottom (Chromabond, Macherey-Nagel, Düren, Germany) and another PE frit at the top to allow the loading of the sample uniformly. The dimensions of the column used in this work were 1.5 cm diameter and 5.6 cm height. The void volume was 2.5 mL determined using dextran blue. Bovine serum albumin (BSA, Sigma, Burbank, CA, USA) was used to check the elution profile of the SEC column. Another column of 5 mL volume was prepared similarly. After chromatography, the column was cleaned by 10 volumes of elution buffer followed by 1 volume 1% (*v/v*) Triton, 1 volume of 0.5 M NaOH, and 10 volumes of elution buffer before reuse.

#### 2.3.2. Size-Exclusion Chromatography of Tomato Fruit-Derived Vesicles

NVs or MVs isolated using dUC and suspended in appropriate volume of extraction buffer (i.e., 500 µL for the 10 mL column and in 250 µL for the 5 mL volume column) were loaded on the SEC column. A total of 30 fractions (500 µL volume each in the case of 10 mL column and 250 µL volume each in case of the 5 mL volume column) were collected by using extraction buffer for elution. Protein quantity in each fraction was measured using Qubit protein assay (Invitrogen, Life Technology Corporation, Eugene, OR, USA). NVs isolated by dUC were mixed in a 1:1 ratio with BSA to determine the efficiency of the separation of vesicles from medium molecular mass soluble proteins.

### 2.4. Characterization of MVs and NVs

For the physicochemical and morphological characterization of NVs and MVs, we applied DLS, NTA, and TEM techniques.

#### 2.4.1. Dynamic Light Scattering

Samples (0.33 mg/mL protein concentration) were centrifuged at 1000× *g* for 10 minutes, and were transferred by using clean pipettes into a quartz cell for DLS measurements. The intensity autocorrelation function g2(t) was measured at 20 °C by using a Brookhaven instrument BI-9000 correlator and a solid-state laser tuned at 532 nm. The autocorrelation function was fit to obtain the distribution of the diffusion coefficients by using the expression *g*_2_(*t*) − 1 = *β* [*∫* *P*(*D*) exp(−*Dq*^2^*t*) *dD*]^2^ and assuming a multi-peak Schultz distribution [33]. Therefore, the size distribution function, namely, the hydrodynamic diameter distribution function P(D_h_), was derived from the distribution of diffusion coefficients P(D) by using the Stokes–Einstein relation *D_h_* = *k_B_T*/(3*πηD*).

#### 2.4.2. Nanoparticle Tracking Analysis

Particle number concentration was measured by NTA using a Nanosight NS300 (Malvern Panalytical, UK). The samples were diluted to obtain less than 100 particles per frame; then, 5 × 60 second measurements were performed with a moderate flow and analyzed by the built in software of the instrument (NanoSight NTA software 3.4 version 003).

#### 2.4.3. Transmission Electron Microscope (TEM)

TEM analyses were performed on NVs and MVs isolated by dUC. Briefly, 5 μL samples at 1 μg/μL protein concentration in 0.1 M PBS (pH 7.6) were deposited onto the formvar and carbon-coated 300 mesh copper grids. After 1 minute, the droplets were removed and the grids were dried. Samples were negatively stained with 2% (*w/v*) aqueous uranyl acetate. TEM images were acquired using a Jeol JEM 1011 electron microscope operating at 60 kV and mounted with a Morada CCD camera (Olympus Soft Imaging Solutions, Münster, Germany).

#### 2.4.4. Protein Profiling by SDS-PAGE

Protein profiles of the different vesicle isolates were obtained by SDS-PAGE. Samples (10 μg of protein based on Qubit protein assay) were electrophoretically separated under reducing conditions on a precast Novex Bolt 4–12% Bis-Tris Plus gel (Invitrogen, Carlsbad, CA, USA) using the Bolt MOPS SDS running buffer (Invitrogen, Carlsbad, CA, USA) according to the manufacturer’s instructions. Gels were stained with colloidal Coomassie blue (Applichem GmbH, Darmstadt, Germany).

#### 2.4.5. Lipid Profiling by Thin-Layer Chromatography

Total lipid extraction and TLC analysis were performed on dUC-isolated NV and MV samples. A total of 200 µg of sample (expressed in protein amount measured by the Qubit protein assay) (Invitrogen, Life Technology Corporation, Eugene, OR, USA) was mixed with 1 mL of methanol/water/chloroform (2.5:1:1) solution at −20 °C. After rigorous mixing for 1 minute, the sample was centrifuged at 15,000× *g* for 5 minutes at 4 °C. Supernatant was collected and pellet was dissolved in 0.5 mL methanol/chloroform (1:1), kept at −20 °C, and centrifuged again at 15,000× *g* for 5 minutes at 4 °C. The resulting 2 supernatants containing the lipid extracts were combined and 300 µL of water was added; then, the sample was centrifuged at 15,000× *g* for 5 minutes at 4 °C. The bottom layer was collected and dried under the stream of nitrogen. The dried sample was suspended in 50 µL of chloroform, and 10 µL was applied on the TLC plate. Lipids were separated on silica gel 60 F254 TLC plates (Merck KGaA, Darmstadt, Germany) by using chloroform/methanol/water (5/1.5/0.5, *v/v/v*) as mobile phase. After development, plate was dried at room temperature. For visualization, the plate was placed in a solution containing 10% copper sulfate (Carlo Erba, Milano, Italy), 8% phosphoric acid (Deltek, Pozzuoli, Italy), and 5% methanol (Romil, Deltek, Pozzuoli, Italy) for 10 seconds, and then the plate was placed in an oven at 150 °C for 10 minutes. Phospholipid (PL) standards, phosphatidylserine (PS), phosphatidic acid (PA), phosphatidylglycerol (PG), phosphatidylcholine (PC), and phosphatidylethanolamine (PE) from Larodan AB (Solna, Sweden) were used for the identification of the lipids. Retardation factors (RFs) of the PL standards were measured in 3 experiments and they were as follows: Rf(PS): 0.21 ± 0.01, Rf(PA): 0.30 ± 0.01, Rf(PG): 0.33 ± 0.01, Rf(PC): 0.40 ± 0.01, and Rf(PE): 0.5 ± 0.01. PLs in the NV and MV samples were tentatively identified on the basis of their RF values. Quantification of the major PLs in NVs and MVs was performed by image analysis scanning densitometry using a VersaDoc (Bio-Rad Laboratories Inc., Munchen, Germany) imaging system in densitometry mode. Experiments were performed 5 times on each sample; mean values and standard deviations were calculated and reported.

#### 2.4.6. Proteomic and Bioinformatics Analysis

Two biological replicates of both the MV and NV fractions isolated and purified by dUC/SEC were subjected to in-solution digestion and nanoLC–MS/MS analysis. Vesicles were lysed using 5 freeze–thaw cycles in the presence of Rapigest (Waters, Milford, MA, USA) detergent according to the manufacturer recommendations. Lysed vesicles were in-solution digested using Trypsin (Mass Spec grade, Promega Corporation, Madison, WI, USA) at a 1:100 ratio. A total of 1 µg tryptic digest was analyzed using a Dionex Ultimate 3000 nanoRSLC (Dionex, Sunnyvale, CA, USA) coupled to a Bruker Maxis II mass spectrometer (Bruker Daltonics GmbH, Bremen, Germany) via CaptiveSpray nanobooster ion source. Samples were desalted by 0.1% trifluoroacetic acid at a flow rate of 5 µL/min for 8 minutes using an Acclaim PepMap100 C-18 trap column (100 µm × 20 mm, Thermo Scientific, Sunnyvale, CA, USA). Peptides eluting from the precolumn were separated on the ACQUITY UPLC M-Class Peptide BEH C18 column (130 Å, 1.7 µm, 75 µm × 250 mm, Waters, Milford, MA, USA) at 300 nL/min flow rate at 48 °C column temperature using a linear gradient from 4% B to 50% B in 120 minutes. Solvent A was 0.1% formic acid, solvent B was acetonitrile with 0.1% formic acid. The cycle time for data-dependent acquisition, was 2.5 s. MS spectra were acquired at 3 Hz, while MS/MS spectra were acquired at 4 or 16 Hz, depending on the intensity of the precursor ion. Singly charged ions were excluded from the analysis. The default peak-picking settings were used to process the raw MS files in MaxQuant version 1.5.3.30 for label-free quantitation. Peptide identifications were performed within MaxQuant using its built-in Andromeda search engine. During the Andromeda search, proteins were examined against a focused database. The focused database was created following a Byonic (v3.6.0, Protein Metrics Inc, Cupertino, CA, USA) search on the merged mgf files against the *Solanum lycopersicum* L. taxonomy (Tax ID: 20161018) using loose criteria (20 ppm precursor and fragment ion mass tolerance, 2% false discovery rate (FDR), two missed cleavages, cysteine as fixed modification, with methionine oxidation and asparagine and glutamine deamidation as variable modifications). In terms of creating the fasta file for the Andromeda search, only active proteins remained, decreasing the number of identified proteins from around 1000 to 200. Only proteins with at least 2 identified peptides were accepted. For the list of active proteins identified and MaxQuant LFQ values (average of the 2 biological replicates), see Table S1. The Gene Ontology (GO) annotation of the top 20 proteins was performed with Perseus v1.6 using the publicly available *Solanum lycopersycum* L. database. The comparison of the protein content of the different types of vesicles was performed and visualized by the Venny 2.1.0 web app (https://bioinfogp.cnb.csic.es/tools/venny/).

## 3. Results and Discussion

**Figure 1:**
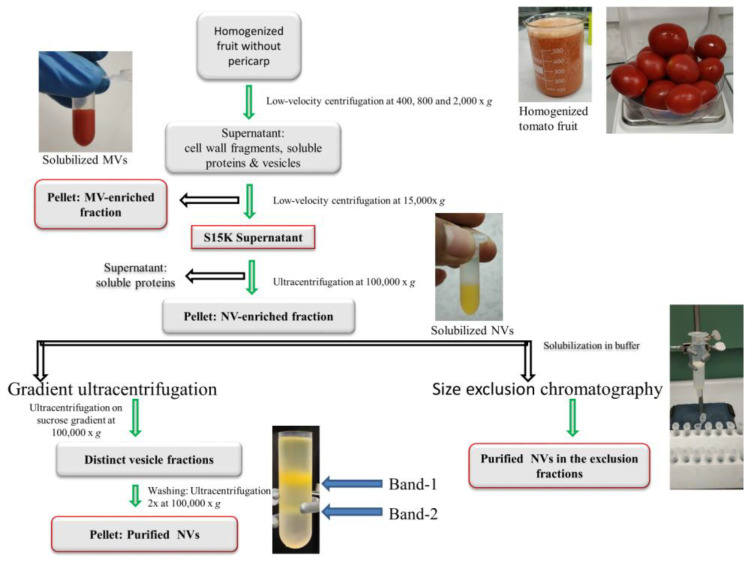
Schematic of the workflow used for the isolation and purification of microvesicles (MVs) and nanovesicles (NVs) from tomato fruit. Differential ultracentrifugation (dUC) was used to prepare samples enriched in MVs and NVs. Further separation and purification of dUC-isolated samples were performed by gradient ultracentrifugation (gUC) or size-exclusion chromatography (SEC). gUC resulted in two visible bands indicated as Band-1 and Band-2 in the insert. A total of 30 SEC fractions were collected in each sample.

**Figure 2:**
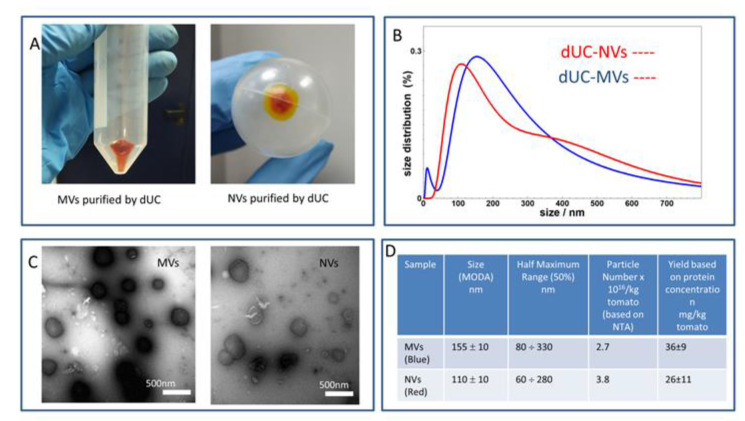
Physical and morphological characteristics and yields of isolated microvesicles (MVs) and nanovesicles (NVs). (**A**) The 15,000× *g* (left image, MVs) and 100,000× *g* centrifugation steps (right image, NVs); (**B**) size distribution of crude MVs (blue line) and NVs (red line) measured in dynamic light scattering (DLS) experiments; (**C**) transmission electron microscopy (TEM) images of MVs and NVs; and (**D**) summary of the results on yield expressed both in protein amounts measured by the Qubit assay and particle numbers measured by nanoparticle tracking analysis (NTA) for kilogram of tomato fruit, and the size (MODA) measured by DLS.

MVs and NVs were isolated from tomato fruit (Piccadilly variety) as red color pellets obtained by dUC after the 15,000× *g* and 100,000× *g* centrifugation steps, respectively (Figure 1). The isolates (Figure 2A) were suspended in the extraction buffer and further purified by (i) gUC using sucrose gradient or (ii) SEC using Sepharose CL-2B-packed SEC columns (Figure 1). Since SEC has not yet been applied to the purification of plant-derived vesicles, we herein aimed to compare the performance of SEC to the more frequently used gUC method.

### 3.1. dUC Isolation and Characterization of Tomato-Derived MVs and NVs

Figure 2 shows the physical characteristics of the crude vesicles obtained by dUC such as size distribution determined by DLS (Figure 2B); vesicle-like morphology acquired by TEM (Figure 2C); and particle and protein concentrations measured by NTA and the Qubit assay, respectively (Figure 2D). In the region below 200 nm, DLS showed size distributions peaked at 110 ± 10 nm and 155 ± 10 nm for NVs and MVs, respectively. In general, the size distributions of MVs and NVs were found to be quite similar. While NVs are on average smaller than MVs, one can notice the presence of a larger size vesicle population in NV samples as well as a population of smaller vesicles in the MV samples. In the case of MVs, we were able to clearly observe a population of very small sized objects, likely freely diffusible proteins (Figure 2B). TEM provided images of objects of vesicle appearance in the size range of 50–500 nm in both samples (Figure 2C). DLS, NTA, and TEM analyses confirmed the presence of heterogeneous vesicle-like objects with different shapes and broad size distribution that are typical characteristics of this kind of complex sample. The presence of small-sized vesicles in the NVs but not in the MVs was expected. In the MVs samples, this could have been due to their co-sedimentation during the 15,000× *g* centrifugation step or to the lysis of the larger vesicle containing organelles such as the chromoplast that are in high quantity in tomato fruit preparations. To determine protein concentration, we used two different assays, the micro bicinchoninic acid assay (BCA assay), which is widely used in EV research, and the Qubit assay. The latter utilizes target-selective dyes that emit fluorescence when bound to proteins. We found that the BCA assay, which is widely used in the determination of EV and NV protein concentration, overestimated the protein concentration in tomato-derived MVs and NVs samples by roughly one order of magnitude in comparison with the Qubit assay and on the basis of the protein profiles obtained by SDS-PAGE using Coomassie blue staining (not shown). Therefore, we show the results of Qubit assay throughout this work. The quantity of vesicles (based on protein concentration) isolated from one kilogram of tomato fruit and measured in three independent extractions (Table in Figure 2D) were 35.6 ± 8.6 mg proteins for MVs and 25.8 ± 11.4 mg proteins for NVs. The particle concentrations measured by NTA were 2.7 × 10^16^ particles for MVs and 3.8 × 10^16^ particles for NVs per 1 kg of tomato fruit. Ratio of particle to protein can be used as a mean of comparing sample purity [34], and these were 8.5 × 10^11^ vesicles/µg of protein and 2 × 10^12^ vesicles/µg of protein for MVs and NVs, respectively. This value for mammalian cell-derived EVs is usually in the range of 10^9^. The higher particle-to-protein ratios for MVs and NVs may indicate that tomato vesicles contain less protein per vesicles than mammalian-cell derived EVs.

**Figure 3:**
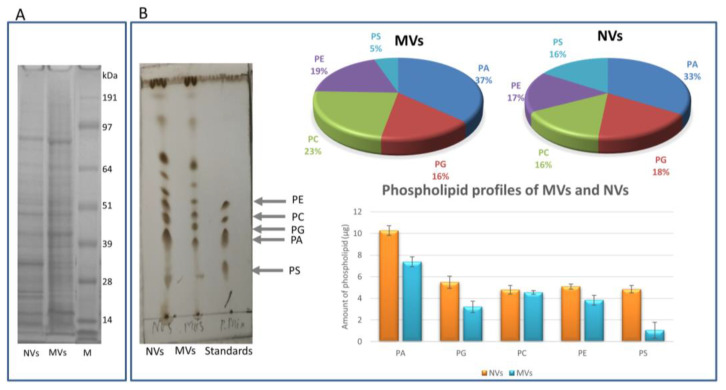
Tomato fruit-derived microvesicles (MVs) and nanovesicles (NVs) showed complex protein and phospholipid profiles. Proteins and lipids were extracted from MV and NV samples isolated by the differential centrifugation (dUC) method. Representative images of (**A**) protein and (**B**) phospholipid (PL) profiles. Phosphatidylserine (PS), phosphatidic acid (PA), phosphatidylglycerol (PG), phosphatidylcholine (PC), and phosphatidylethanolamine (PE) were used as standards for the tentative identification of PLs in 200 µg (expressed as protein quantity measured in the Qubit assay) of NVs and MVs (panel (**B**) left image). Relative (upper right image in panel (**B**)) and absolute PL quantitates (lower right image in panel (**B**)) were determined by densitometric image analysis scanning.

SDS-PAGE (Figure 3A) and TLC analyses (Figure 3B) confirmed the complex protein and lipid contents, respectively, a typical characteristic of phospholipid-enclosed vesicles. The SDS-PAGE protein profiles (Figure 3A) of MVs and NVs were similar but not identical. In both samples, we could observe numerous bands distributed evenly throughout the gel. PLs are the major lipids in plant-derived vesicles. Here, we extracted lipids from 200 µg of vesicles (expressed in protein content) from MVs and NVs and analyzed their PS, PA, PG, PC, and PE contents on the basis of their Rf and intensities values measured in the TLC densitometry analyses in five replicates (Figure 3B). The PL profiles of tomato-derived MVs and NVs were similar. Relative and absolute amount of PLs are reported in Figure 3B. NVs contained more PLs (30 µg) than MVs (20 µg). Similarly to ginger- [20] and grape-derived vesicles [16], the most abundant PL component was PA in both MVs (7.4 ± 0.5 µg) and NVs (10.3 ± 0.4 µg). A more than four times higher relative amount of PS was measured in NVs (4.8 ± 0.4 µg) in comparison with MVs (1 ± 0.7 µg). At high Rf, values other than phospholipids were also observed (Figure 3B left side image) on the TLC plate.

### 3.2. Gradient Ultracentrifugation of NVs Isolated by dUC

**Figure 4:**
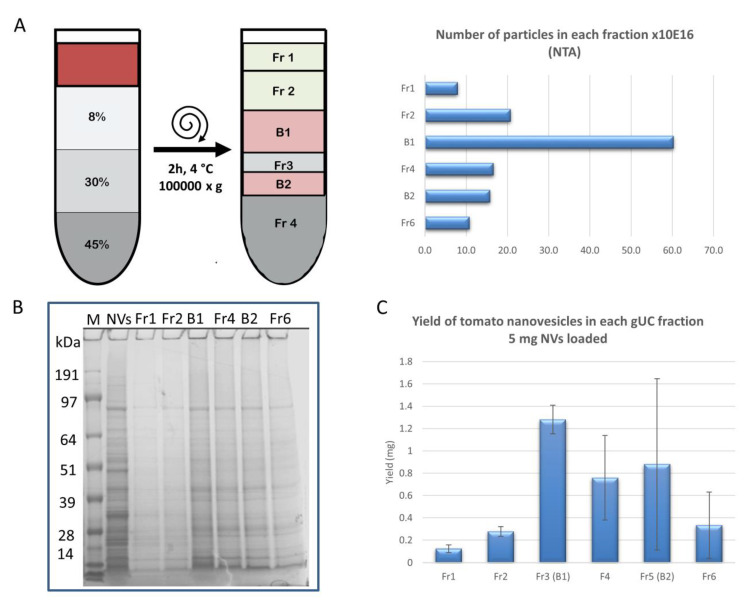
Tomato fruit-derived nanovesicles (NVs) isolated and separated by the differential ultracentrifugation (dUC) method followed by gradient ultracentrifugation (gUC) using 8%/30%/45% (w/v) stepwise sucrose gradient. dUC/gUC after 2 hours of centrifugation at 100,000× *g* yielded two visible bands, as shown in Figure 1: band 1 (B1) and band 2 (B2). Six fractions (F1, F2, B1, F3, B2, and F6) were collected and analyzed. (**A**) Results of nanoparticle tracking analysis (NTA) showing the number of particles in each fraction, (**B**) proteins of each fraction were separated by sodium dodecyl sulphate polyacrylamide gel electrophoresis (SDS-PAGE), and (**C**) yield expressed in protein amount calculated on the basis of Qubit assay in each fraction.

The NV sample was subjected to gUC using a stepwise 8/30/45% (*w/v*) sucrose gradient to remove co-purifying impurities associated with the vesicles and to separate the crude NVs into discrete EV-like fractions on the basis of density in three extraction experiments using different starting NV samples. Densities of mammalian EVs in sucrose were in the range of 1.13–1.19 g/mL that is obtainable at the 30–40% (*w/v*) sucrose concentration range. The protocol applied is similar to that previously used for ginseng vesicles [21]. Six fractions were collected from top to bottom. Two of the fractions contained visible bands: B1 (corresponds to Fraction 3) at low-density and B2 (corresponds to Fraction 5 in Figure 1 and Figure 4A) at high density. SDS-PAGE protein profiles of the crude NV isolated by dUC and the density-separated six fractions are shown in Figure 4B.

SDS-PAGE protein profiles of the gUC-separated NV fractions showed less background level and more resolved protein bands when compared to the dUC NVs sample. NTA confirmed the presence of particles in each gUC fraction. Distribution of particle numbers in the six fractions is shown in (Figure 4A). The highest number of particles were concentrated in B1. The separation of dUC-isolated NV sample into discrete vesicle populations indicates that this isolate contained vesicles not only of different shapes (as confirmed by TEM) and sizes (as confirmed by DLS) but also of different densities. Protein quantity obtained in each fraction is shown in Figure 4C. This dataset is related to the same experiment when NTA (Figure 4A) and SDS-PAGE (Figure 4B) were performed. The mean (*n =* 3) yields for the two visible bands were as follows: B1: 1.28 ± 0.13 mg, and B2: 0.88 ± 0.77 mg. The sum of these two bands corresponded to 43.2% of the total protein amount loaded (5 mg). We found that the relative protein amounts in these two bands were highly variable in the different experiments, and probably depended on the quality of the fruit used for the extraction.

### 3.3. SEC Purification of MVs and NVs Isolated by dUC

SEC was performed using a gravity column packed with Sepharose Cl-2B (Figure 1) and equilibrated with the extraction buffer to purify the crude NVs and MVs isolated by dUC. Two columns with bed volumes of 10 and 5 mL were packed to evaluate the influence of column bed volume on the separation and loading capacity. The effect of exogenously added soluble proteins on separation efficiency and the presence of vesicles in the supernatant obtained in the 15,000× *g* centrifugation step (Figure 1) were also studied.

**Figure 5:**
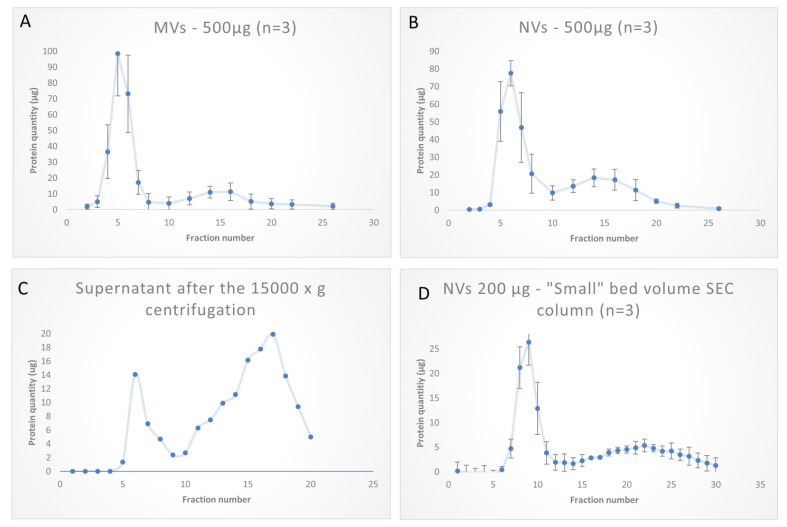
Tomato fruit-derived microvesicles (MVs) and nanovesicles (NVs) purified by size-exclusion chromatography (SEC); 30 fractions were collected in each sample. SEC chromatograms show the protein quantities determined in each fraction by the Qubit assay. (**A**) MVs pelleted after the 15,000× *g* centrifugation step; (**B**) NVs obtained as pellet after the 100,000× *g* centrifugation step; (**C**) supernatant after 15,000× *g* centrifugation step using a 10 mL bed volume SEC gravity column; (**D**) 5 mL bed volume columns were used to separate 200 µg of NVs.

MVs or NVs isolated by dUC were loaded on the SEC column and 30 volume fractions (0.5 mL) were collected. Vesicles and large molecules cannot enter the pores and are eluted in the column’s void volume. Protein concentrations and particle numbers were measured and evaluated in each fraction. We obtained chromatograms with two broad peaks in both MVs and NVs (Figure 5A,B)—the first most abundant peak was eluted at fractions 4–6, and a second smaller and broader peak at fractions 15–17 (Figure 5C). The highest protein concentration was measured at fraction 5. These elution profiles indicate that most of the proteins were associated with the vesicles, and only a smaller portion of the signal could be attributed to the co-purifying proteins in the sample.

**Figure 6:**
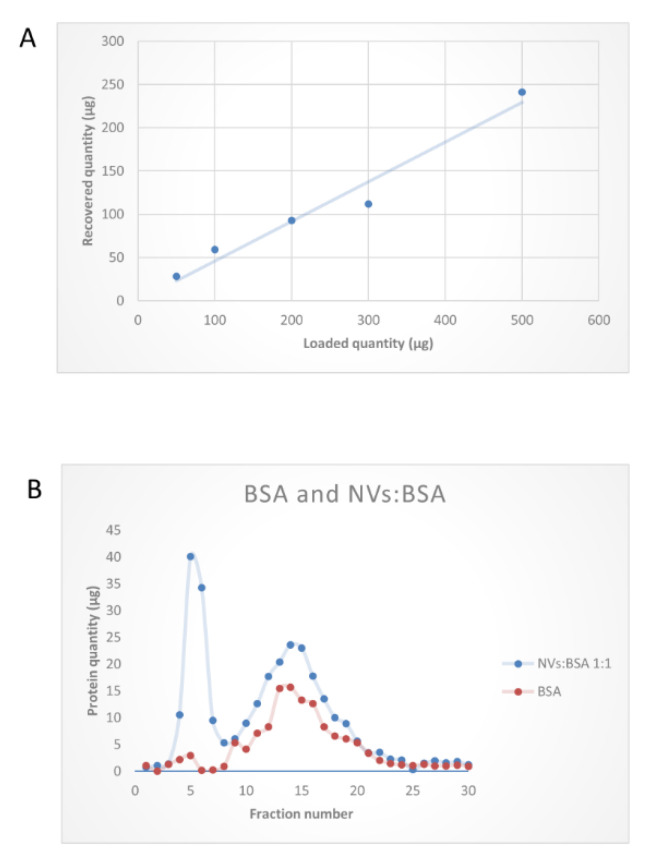
Performance of size-exclusion chromatography (SEC) in the purification of tomato-derived nanovesicles (NVs) isolated by differential ultracentrifugation. (**A**) Graph shows the quantities of SEC-purified NVs obtained at increasing loading quantities (50, 100, 200, 300, and 500 µg expressed in protein amounts) and (**B**) SEC chromatograms of bovine serum albumin (BSA) protein standard (in red) and a 1:1 mixture of NVs and exogenously added BSA (in blue) showing the separation efficiency of the SEC.

**Figure 7:**
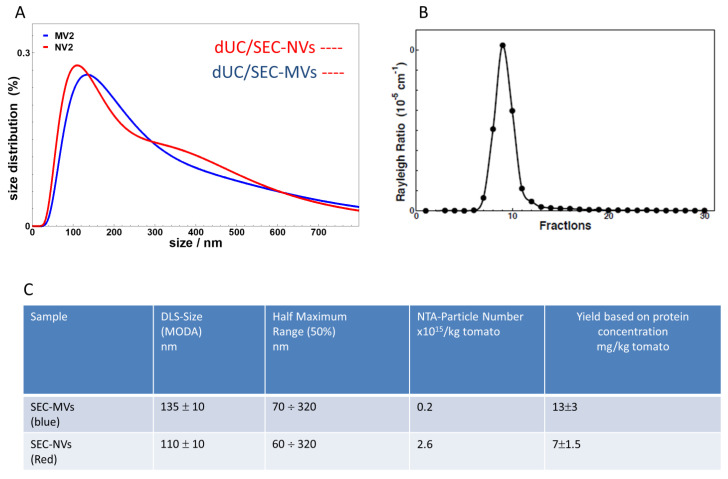
Physical characteristics and yields of microvesicles (MVs) and nanovesicles (NVs) isolated using the differential ultracentrifugation (dUC) method and purified by size-exclusion chromatography (SEC). (**A**) Size distribution measured by dynamic light scattering (DLS) in the pooled 4–6 SEC fractions. (**B**) Rayleigh ratio measured in each SEC fraction and (**C**) DLS moda, nanoparticle tracking analysis (NTA) particle size, and protein yields measured in the pooled 4–6 SEC fractions.

The performance of SEC column was tested at varying loading quantities in the range of 50–500 µg. (Figure 6A) shows a linearly increasing yield with the increasing loading amounts in the studied range. Further, to prove the utility of SEC in removing exogenous soluble proteins from the vesicle samples, we performed experiments in which 200 µg bovine serum albumin (BSA) alone or mixed 1:1 with NVs were loaded. BSA in these experiments represented the soluble protein exogenously added to the vesicles. Figure 7B demonstrates that SEC efficiently separated the vesicles (fractions 4–6) from BSA, which eluted in later fractions (fractions 10–20), confirming a good efficiency in separation. SEC-based purification of NVs was performed using a smaller (5 mL) bed volume column with different quantities. One example is shown in Figure 5D when 200 µg of crude NVs were purified in three consecutive runs. Elution profiles of NVs obtained using the two columns with different bed volumes (10 mL and 5) were very similar. In one experiment, we loaded the filtered (0.45 µm followed by a 0.22 µm pore size filters) supernatant after the 15,000× *g* centrifugation step (S15k); thus, instead of the ultracentrifuge, SEC was used to isolate NVs directly. The chromatogram (Figure 5C) showed a peak (with a maximum at fraction 6) in the exclusion volume, which is typical of NVs. The calculated total amount of NVs purified by SEC from the S15K fraction was slightly lower than that obtained by the dUC/SEC purification.

**Figure 8:**
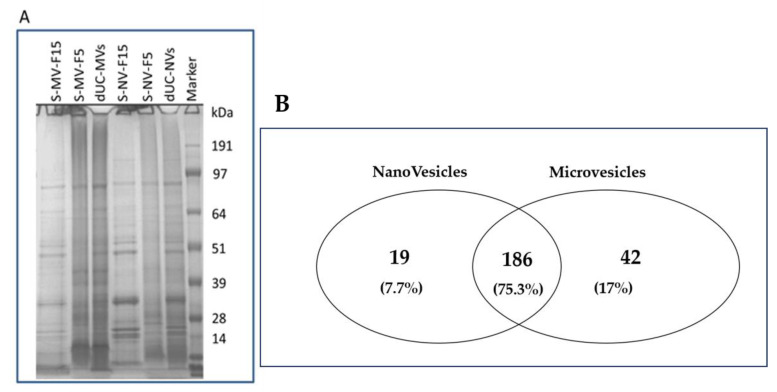
Protein characterization of nanovesicles (NVs) and microvesicles (MVs) isolated by differential ultracentrifugation (dUC) and purified using size-exclusion chromatography (SEC). (**A**) Sodium dodecyl sulphate polyacrylamide gel electrophoresis (SDS-PAGE) image shows the protein profiles of two SEC-separated fractions (F5 and F15) of NVs and MVs and (**B**) Venn diagram of the proteins identified in the proteomics study (refer to Table 2 and Table S1 for the full set of proteins identified and quantified) showing a high level of similarity between the identified protein sets in MVs and NVs.

Fractions 4, 5, and 6 were combined and analyzed by DLS, NTA (Figure 7), SDS-PAGE, and MS-based proteomics (Figure 8). Figure 7A shows the intensity average distribution measured by DLS with a maximum (or mode) at 110 ± 10 nm and 135 ± 10 nm for NVs and MVs, respectively. There was not much difference in the size distribution of NVs before and after SEC purification, but we observed that the size distribution of MVs was slightly changed, as we found nearly 20 nm decrease in the moda of MVs. This may have been due to the entrapment of the larger vesicles into 2% cross-linked agarose gel filtration resins. In addition, the fraction of small-size objects, likely free proteins, detected in the crude dUC-isolated MV and NV samples (Figure 2B) were removed by SEC. The Rayleigh ratios were measured in the 30 SEC fractions of NVs isolated using the small bed volume column (SEC chromatogram is shown in Figure 7B), and they were found to be proportional to the average molecular mass times the mass concentration. Thus, they directly represented the amount of particles purified in each sample. More specifically, in this case, since we were dealing on average with the same nanoparticles, the Rayleigh ratio profile as a function of sample fraction could be taken as a measure of particle concentration.

From NTA data, we found 0.2 × 10^15^ and 2.6 × 10^15^ particles for 1 kg of tomato fruit, respectively (Figure 6). Regarding the yields, after SEC purification, we obtained 13.1 (MVs) and 6.9 mg vesicles (NVs) per kilogram of tomato fruit on the basis of the protein content measured. Particle to protein ratios were 1.7 × 10^11^ vesicles/µg of protein and 4.6 × 10^11^ vesicles/µg of proteins for MVs and NVs, respectively.

SEC-purified vesicles showed less complex proteins patterns than the bulk NVs and MVs in the SDS-PAGE image (Figure 8A). SEC was proven to be efficient in removing small and medium molecular mass proteins from the vesicle sample, as can be appreciated in the SDS-PAGE image of the late eluting fractions (S-MV-Fraction 15 and S-NV Fraction 15 in Figure 8A). To gain further insights into the composition of the protein biocargo of the SEC-purified vesicles, we performed proteomics analysis (Section 3.2).

### 3.4. Proteomic Characterization of dUC/SEC-Purified MVs and NVs

Mass spectrometry (MS)-based proteomics was performed to gain insights into the protein cargo of tomato-derived NVs and MVs. MS-based downstream analysis requires highly purified samples; therefore, only SEC-purified MVs and NVs were analyzed. Vesicle samples were lysed and digested by trypsin in-solution prior to nanoHPLC–electrospray ionization (ESI)–MS analysis. A total of 228 proteins were identified in the MV fraction and 205 proteins in the NV fraction (Table S1). Proteomic profile of the SEC-purified NVs and MVs was similar as 75.3% of the identified proteins were detected in both vesicle types (Figure 8B, Venn diagram).

Protein quantification was carried out using label-free proteomics. The 20 most abundant proteins are listed in (Table 2), together with their cellular localization. Of the 20 most abundant proteins, 16 were present in both the MVs and NVs. Most of the top-ranking proteins were enzymes. The most abundant protein in both fractions was lipoxygenase (LOX). Lipoxygenases are a family of enzymes that dioxygenate unsaturated fatty acids and thus participate in oxylipin biosynthesis. LOXs are involved in plant growth and development, ripening, and plant defense mechanisms. Plant LOXs have found some applications in the green food additives’ industry as strengthening and bleaching agents [42]. Adenosine triphosphate (ATP) synthase (ATPA and ATPB) and heat shock proteins (HSP70, HSP80 and G5DGD4) have been reported to be highly expressed in other plant-derived vesicle preparations [27]. Amongst the 20 most abundant proteins, several are involved in fruit ripening, e.g., ACCH3_SOLLC, ASR1_SOLLC, and PGLR_SOLLC. Several proteins identified only in the MV sample are localized in subcellular organs such as the mitochondrion (SUCB_SOLLC, B1Q3F8_SOLLC, and Q8GT30_SOLLC). Glucan endo-1,3-beta-glucosidase B, a vacuolar protein detected in the MV sample, participates in defense against pathogens. The NVs also contained proteins that play a role in plant defense such as osmotin-like protein. Our results were compared to a recent article focusing on the proteomics analysis of chromoplast from six crop species including tomato [43]. Tomato fruit is composed of several different tissue types. The pericarp formed from the ovary wall and the placenta comprise the fleshy tissue of the tomato—mainly a pericarp, the locular tissue, and the seeds [44]. During fruit ripening, chloroplasts differentiate into photosynthetically inactive specialized plastids, called chromoplasts, which are present in relatively high amount in tomato fruit. Chromoplasts are where the colored carotenoids are synthetized and accumulate. Chromoplasts are a double lipid bilayer-surrounded structure of about 1 µm in diameter, and they are isolated using the gUC-based method. Interestingly several abundant proteins identified in chromoplasts were also detected in both the MV and NV fractions, suggesting a possible chromoplast origin of some of the vesicles in our preparations. These proteins were alcohol dehydrogenase 2, ATP synthase subunit alpha and beta, and adenosine diphosphate (ADP) adenosine triphosphate (ATP) translocator.

## 4. Conclusions

Conventional dUC isolates an average 0.5–1.5 × 10^13^ particles from 1 liter of mammalian cell culture medium or 3–4 µg protein per 10^6^ cells [45]. Plant-derived EV-like vesicles can be produced at a considerably higher yield, mainly because they contain a mixture of different populations of intra- and extracellular vesicles. [27] Ginger, for example, is one of the most promising edible resources of NVs. Ginger-derived vesicles show high antinflammatory activity and they can be produced at a high yield [6,9,19,36,40]. Chen et al. isolated 0.5–2 ×10^14^ particles per kilogram of ginger root using the gUC method [17], which is about 10 times more than that obtainable in current mammalian production systems. Fruit and fruit juices are also valuable sources for the isolation of EV-like vesicles (Table 1). While grapefruit and lemon have been studied by several authors, tomato fruit has been less exploited thus far (Table 1).

Recently, Wang et al. reported the isolation of tomato-derived vesicles using the dUC/gUC method and obtained 440 ± 20 mg vesicles per kilogram of fruit [25]. However, this yield was based on the measured weight of the obtained NV sample. Different to this, we herein calculated the yield on the basis of commonly used methods for the quantitation of EVs (47) i.e., protein concentration and the number of particles determined by NTA. Isolating the vesicles by the classical dUC method, we obtained exceptionally high yields with high particle to protein ratios: 2.7 × 10^16^ MVs (35.6 ± 8.6 mg protein) and 3.8 × 10^16^ (25.8 ± 11 mg protein) NVs particles (Figure 2D). We found that the number of isolated NVs was higher than that of MVs, yet the protein content was lower. This may indicate a higher presence of liposome-like vesicles in the NVs with respect to MVs. Vesicle isolates were analyzed using DLS, NTA, TEM, SDS-PAGE, and TLC. Interestingly, we found no significant differences between the size distribution, morphology, and molecular content of tomato-derived MV and NV isolates. These isolates were further purified and separated using SEC or gUC. Through SEC (Figure 1; Figure 5, Figure 6 and Figure 7), we purified 27% of the loaded crude MVs and 37% of NVs as pure nano-sized vesicular objects. On the other hand, gUC yielded 25.6% and 17.6% of the loaded crude NVs in the two visible bands, B1 and B2, respectively (Figure 4). When comparing the performance of two methods, gUC was proven to be more useful in the separation of different vesicle populations (on the basis of buoyant density) in comparison with SEC. Instead, SEC was efficient in the removal of the co-purifying proteins and other impurities, and thus improved the quality of NV and MV preparations and enabled them for subsequent downstream analysis, such as omics (Figure 8 and Table 2) and biological characterizations. We should point out that both methods required concentrated samples for the separation. Proteomic and lipidomic analysis revealed the main proteins (lipoxygenase, alcohol dehydrogenase 2, protein E8, ATPases, and abcsistic stress protein 1) and phospholipids (PA, PS, PG, PE, and PC) in these isolates. Since edible plant-derived vesicles can be found at relatively low concentrations in highly diluted samples, their isolation enrichment by dUC, TFF, precipitation, or other means is necessary. We found SEC to be easy to perform.

Summarizing our results, we made a step forward in the purification of MVs and NVs from plant resources by introducing a SEC purification step after the dUC separation with the aim of improving the purity of the vesicle isolates for downstream applications.

## Figures and Tables

**Table 1:** Methods used for the isolation of plant-derived microvesicles (MVs), nanovesicles (NVs), and apoplastic vesicles (AVs) from different organs, such as fruit, flower, seed, rhizome, and leaf, and the yields obtained.

Resource	Organ	Isolation Method	Vesicle Type(s) Isolated	Yield (g/L or g/kg of Starting Plant Material)	Particle Number (Particles/kg or Particles/L of Starting Plant Material)	Ref.
Ginger	rhizome	dUC/gUC	NVs	0.25–1.25 g/L	n.r.	[21]
Ginger	rhizome	dUC/precipitation	NVs	2-3.8	n.r.	[22]
Ginger	rhizome	dUC	NVs	4	n.r.	[22]
Ginger	rhizome	dUC	NVs	48.5 ± 4.8 × 10^−3^	n.r.	[20]
Ginger	rhizome	dUC/gUC	NVs	0.890	4.2 × 10^12^	[23]
Ginger	rhizome	dUC	NVs	n.r.	0.5 − 2 × 10^14^	[17]
Ginger	rhizome	dUC/gUC	NVs	Three bands each containing ≅0.05	n.r.	[13]
Grape	fruit	dUC/gUC	NVs	1.76 ± 0.15	n.r.	[19]
Grapefruit	fruit	dUC/gUC	NVs	2.21 ± 0.044	n.r.	[19]
Tomatoes	fruit	dUC/gUC	NVs	0.44 ± 0.02	n.r.	[19]
Grape	fruit	dUC/gUC	NVs	n.r.	n.r.	[16]
Broccoli	flower	dUC/gUC	NVs, MVs	n.r.	n.r.	[14]
Apple	fruit	dUC	NVs	n.r.	1.6 × 10^13^ particles/L	[24]
Coconut	fruit	dUC/MF	NVs	n.r.	n.r.	[25]
*Citrus clementina*	fruit	dUC/gUC	NVs	1.67 × 10^−3^ g/L (protein)	1.16 × 10^12^ particles/L	[26]
*Citrus sinensis*	fruit	dUC	NVs	0.178 g/L (protein)	n.r.	[27]
*Citrus paradisi*	fruit	dUC	NVs	0.134 g/L (protein)	n.r.	[27]
*Citrus aurantium*	fruit	dUC	NVs	0.161 g/L (protein)	n.r.	[27]
*Citrus limon*	fruit	dUC	NVs	0.409 g/L (protein)	n.r.	[27]
*Citrus limon*	fruit	dUC/MF/gUC	NVs	2.5 × 10^−3^ g/L	n.r.	[12]
Carrot	root	dUC/gUC	NVs	0.298	n.r.	[28]
Blueberry	fruit	dUC/MF	NVs	n.r.	n.r.	[29]
Hami melon	fruit	dUC/MF	NVs	n.r.	n.r.	[29]
Pea	seed	dUC/MF	NVs	n.r.	n.r.	[29]
Pear	fruit	dUC/MF	NVs	n.r.	n.r.	[29]
Soybean	seed	dUC/MF	NVs	n.r.	n.r.	[29]
Orange	fruit	dUC/MF	NVs	n.r.	n.r.	[29]
Kiwifruit	fruit	dUC/MF	NVs	n.r.	n.r.	[29]
*Arabidopsis thaliana* L.	leaf	dUC/gUC	EVs	n.r.	n.r.	[30]
Sunflower	seed	MF/dUC	AVs	n.r.	n.r.	[31]
*Nicotiana tabacum* L.	leaf	dUC	AVs	n.r.	n.r.	[32]
*Vinca minor* L.	leaf	dUC	AVs	n.r.	n.r.	[32]
*Viscum album* L.	leaf	dUC	AVs	n.r.	n.r.	[32]
*Phaseolus vulgaris* L.	leaf	dUC	EVs	0.081 ± 0.03	n.r.	[33]
*Oryza sativa* L. (Rice)	leaf	dUC	AVs	n.r.	n.r.	[34]

Abbreviations are as follows: dUC: differential ultracentrifugation, gUC: gradient ultracentrifugation, MF: microfiltration, n.r.: not reported data, and Ref.: reference. Yield refers to the weight of the vesicle-containing pellet if not stated otherwise.

**Table 2:** List of the 20 top-ranking proteins in the microvesicle (MV) and nanovesicle (NV) samples isolated and purified by differential ultracentrifugation and size-exclusion chromatography (dUC/SEC) from tomato fruit (Figure 1). For the full set of proteins identified, refer to Table S1.

	UniProtKB	Protein Names	Typical Subcellular Location	NV Intesnsity	MV Intensity
1	Q42873_SOLLC	Lipoxygenase	cytoplasm	2.94 × 10^8^	3.62 × 10^8^
2	ADH2_SOLLC	Alcohol dehydrogenase 2	cytoplasm	1.45 × 10^8^	1.47 × 10^8^
3	ACCH3_SOLLC	1-aminocyclopropane-1-carboxylate oxidase homolog		1.23 × 10^8^	1.16 × 10^8^
4	ATPB_SOLLC	ATP synthase subunit beta, chloroplastic	chloroplast	1.14 × 10^8^	6.02 × 10^7^
5	ASR1_SOLLC	Abscisic stress-ripening protein 1	nucleus	1.04 × 10^8^	1.48 × 10^8^
6	PGLR_SOLLC	Polygalacturonase-2 (Pectinase)	cell wall, apoplast	1.04 × 10^8^	2.04 × 10^8^
7	ATPA_SOLLC	ATP synthase subunit alpha, chloroplastic	chloroplast	9.11 × 10^7^	4.44 × 10^7^
8	EF1A_SOLLC	Elongation factor 1-alpha	cytoplasm	8.20 × 10^7^	9.24 × 10^7^
9	P93767_SOLLC	ADP/ATP translocator	membrane	7.28 × 10^7^	5.78 × 10^7^
10	Q40140_SOLLC	Aspartic protease		6.38 × 10^7^	3.58 × 10^7^
11	Q9XEX8_SOLLC	Remorin 1		6.14 × 10^7^	4.06 × 10^7^
12	H1ZXA9_SOLLC	Heat shock protein 70 isoform 3	cytoplasm	6.09 × 10^7^	9.74 × 10^7^
13	HSP80_SOLLC	Heat shock cognate protein 80	cytoplasm	6.06 × 10^7^	7.05 × 10^7^
14	G5DGD4_SOLLC	Class I small heat shock protein		6.01 × 10^7^	4.57 × 10^7^
15	K4CJ46_SOLLC	2-Isopropylmalate synthase	chloroplast, cytoplasm	5.88 × 10^7^	5.40 × 10^7^
16	B0JEU3_SOLLC	Vicilin		5.81 × 10^7^	4.65 × 10^7^
17	Q4W5U7_SOLLC	Calnexin-like protein	ER	5.74 × 10^7^	4.24 × 10^7^
18	Q6IV07_SOLLC	UDP-arabinopyranose mutase	cytosol/Golgi	5.27 × 10^7^	1.50 × 10^7^
19	G8Z279_SOLLC	Hop-interacting protein THI113		4.54 × 10^7^	4.69 × 10^7^
20	Q38JD4_SOLLC	Temperature-induced lipocalin	cytoplasm	4.50 × 10^7^	3.70 × 10^7^
21	FSPM_SOLLC	Fruit-specific protein		6.51× 10^6^	5.78 × 10^7^
22	O81536_SOLLC	Annexin	cytoplasm	2.92 × 10^7^	5.76 × 10^7^
23	CATA1_SOLLC	Catalase isozyme 1	peroxisome	2.16 × 10^7^	4.42 × 10^7^
24	RS27A_SOLLC	Ubiquitin-40S ribosomal protein S27a	nucleus	3.64 × 10^7^	4.10 × 10^7^

## Supplementary Materials

The following are available online at https://www.mdpi.com/2304-8158/9/12/1852/s1: Table S1: Details on proteomics data title.
